# Orexin and Sleep Disturbances in Alpha-Synucleinopathies: a Systematic Review

**DOI:** 10.1007/s11910-024-01359-6

**Published:** 2024-07-20

**Authors:** Kausar Raheel, Qi Rui See, Veronica Munday, Basma Fakhroo, Olga Ivanenko, Marcello Luigi Salvatelli, Carlotta Mutti, Peter J. Goadsby, Alessio Delogu, Sharon L. Naismith, Phil Holland, Liborio Parrino, K. Ray Chaudhuri, Ivana Rosenzweig

**Affiliations:** 1 Sleep and Brain Plasticity Centre, Department of Neuroimaging, Institute of Psychiatry, Psychology and Neuroscience (IoPPN), King’s College London, De Crespigny Park, London, SE5 8AF UK; 2 Sleep Disorders Center, Department of General and Specialized Medicine, University Hospital of Parma, 43125 Parma, Italy; 3 NIHR-Wellcome Trust King’s Clinical Research Facility, King’s College London, London, WC2R 2LS UK; 4 Basic and Clinical Neuroscience, IoPPN, King’s College London, London, WC2R 2LS UK; 5 Healthy Brain Ageing Program, School of Psychology; Brain and Mind Centre, The University of Sydney, & Charles Perkins Centre, Camperdown, Sydney Australia; 6 Department of Medicine and Surgery, Neurology Unit, University of Parma, 43125 Parma, Italy; 7 Movement Disorders Unit, King’s College Hospital and Department of Clinical and Basic Neurosciences, Institute of Psychiatry, Psychology & Neuroscience and Parkinson Foundation Centre of Excellence, King’s College London, London, UK; 8 Sleep Disorders Centre, Guy’s and St Thomas’ NHS Foundation Trust, London, UK

**Keywords:** Orexin, Sleep Disturbances, Alpha-synucleinopathies, Parkinson's Disease (PD); Dementia with Lewy Bodies (DLB)

## Abstract

**Purpose of Review:**

Sleep disturbances are amongst most frequent non-motor symptoms of Parkinson’s Disease (PD), and they are similarly frequently reported in other alpha-syncleinopathies, such as Dementia with Lewy Bodies (DLB) and Multiple System Atrophy (MSA). More recently, the orexin system has been implicated in control of arousal based on salient environmental set points, and its dysregulation in sleep issues in alpha-synucleinopathies suggested by the findings from the translational animal models. However, its role in the patients with alpha-synucleinopathies remains unclear. We thus set to systematically review, and to critically assess, contemporary evidence on the association of the orexinergic system and sleep disturbances in alpha-synucleinopathies. In this systematic review, studies investigating orexin and sleep in alpha-synucleinopathies (Rapid Eye Movement (REM) Behaviour Disorder (RBD), Parkinson's Disease (PD), Dementia with Lewy Bodies (DLB), Multiple System Atrophy (MSA)) were identified using electronic database searches of PubMed, Web of Science and PsychINFO using MeSH terms, keywords, and title words such as “Alpha-synucleinopathies” AND “Orexin” AND “Sleep Disturbances”.

**Recent findings:**

17 studies were included in this systemic review, of which 2 studies on RBD, 10 on PD, 4 on DLB, and 1 on MSA patients. Taken together, RBD and PD studies suggest a potential adaptive increase in orexin levels in early stages of the neurodegenerative process, with reduced levels more often reported for later, more advanced stages of illness. To date, no differences in orexin levels were demonstrated between MSA patients and healthy controls.

**Summary:**

There is a dearth of studies on the role of orexin levels in alpha-synucleinopathies. Moreover, significant methodologic limitations in the current body of work, including use of non-standardised research protocols and lack of prospective, multi-centre studies, disallow for any finite conclusion in regards to underlying pathomechanisms. Nonetheless, a picture of a complex, multifaceted relationship between the dysregulation of the orexinergic pathway and sleep disturbances in alpha-synucleinopathies is emerging. Hence, future studies disentangling orexinergic pathomechanisms of alpha-syncleinopathies are urgently needed to obtain a more comprehensive account of the role of orexinergic pathway in alpha-synucleinopathies. Pharmacological manipulations of orexins may have multiple therapeutic applications in treatment strategies, disease diagnosis, and might be effective for treating both motor and non-motor symptoms.

**Supplementary Information:**

The online version contains supplementary material available at 10.1007/s11910-024-01359-6.

## Introduction

It has been argued that alpha-synucleinopathies, and in particular Parkinson disease (PD), are amongst the fastest growing neurological disorder worldwide [1]; they are a group of neurodegenerative disorders characterised by pathological hallmarks such as abnormal accumulation of alpha-synuclein positive inclusions in the neurons and/or glial cells that trigger progressive neuronal death through a series of cascading cellular pathways (i.e., mitochondrial impairment, lysosomal dysfunction) [2, 3]. Neurodegenerative diseases that are classified as alpha-synucleinopathies include Parkinson’s Disease (PD), Multiple System Atrophy (MSA), and Dementia with Lewy Bodies (DLB) [2]. All alpha-synucleinopathies appear to exhibit similar clinical, neurochemical, and morphological features and disease pathomechanisms including neuroinflammation [2]. Despite that, variation in clinical phenotypes between and within alphasynucleinopathies has been widely reported, depending on the cellular location and pattern of accumulation [4, 5]. For example, neuronal inclusions, Lewy bodies and Lewy neurites are distinct characteristics for PD and DLB, whilst alpha-synuclein positive glial cytoplasmic inclusions in oligodendrocytes are hallmarks in MSA [2–4, 6].

Patients with alpha-synucleinopathies commonly suffer from significant and debilitating sleep disturbances that include nocturnal and diurnal manifestations such as insomnia, excessive daytime sleepiness, periodic limb movements [7], circadian rhythm abnormalities, poor sleep quality and rapid eye movement sleep behaviour disorders (RBD) (please refer to some relevant studies and disorders listed in Table 1) [15]. For instance, nocturnal sleep disturbances affect 60% to 98% of people with PD [24–26], whilst excessive daytime sleepiness, and other sleep disturbances occur in approximately 80% of DLB patients [13, 15, 27]. Although the underlying pathomechanism of these sleep disturbances remains elusive, numerous preclinical and clinical studies have argued the association of orexin and sleep disturbances in alpha-synucleinopathies [28–31].

**Table 1:** Common Sleep Disorders in Alpha-synucleinopathies

Alpha-synucleinopathy	Sleep Disorder/Problem	Estimated Prevalence
Parkinson’s Disease	REM sleep behaviour disorder (RBD)	50% [8]
	Excessive daytime sleepiness (EDS)	49.7% [9]
	Insomnia disorder	32% [10]
	Nightmare disorder	32% [10]
	Restless legs syndrome (RLS)	20.3% [11]
Dementia with Lewy Bodies (DLB)	REM sleep behaviour disorder (RBD)	89% [12]
	Excessive daytime sleepiness (EDS)	80% [13]
	Nightmare disorder	83% [14]
	Insomnia disorder	26% to 75% [15]
Multiple System Atrophy (MSA)	REM sleep behaviour disorder (RBD)	49.7% to 88% [16–18]
	Sleep fragmentation	52.5% [19]
	Obstructive Sleep Apnea (OSA)	37% [20]
	Restless legs syndrome (RLS)	28% [21, 22]
	Excessive daytime sleepiness (EDS)	27.3% [17]
	Insomnia disorder	19% [23]

Orexin, also known as hypocretin, is a neuropeptide that is predominantly released from orexinergic neurons in the lateral hypothalamus and the perifornical area; it exist in two orexin isoforms, orexin-A and orexin-B [28, 32–34]. Orexin-A acts predominantly on orexin-1 (OX1R) and orexin-2 receptors (OX2R) whilst Orexin-B on the OX2R [28]. There is a widespread and complimentary expression of OXRs receptors in most brain areas, although OX1R are principally expressed in amygdala and locus coeruleus, and OX2R is predominantly expressed in the hypothalamus [28, 35]. Orexin-A and -B are also known to be involved in a number of other functions, including stress-adaptive responses [36], feeding behaviour [37], reward-seeking, drug addiction [38] and even in pathological conditions such as migraines and headaches [39–41]. Importantly, orexin neurons play a central role in promoting wakefulness and stabilising sleep by modulating cholinergic and monoaminergic neurons via strong projections to brain regions involved in the sleep-wake circuitry, such as the locus coeruleus, dorsal raphe nucleus and tuberomammillary nucleus [42], whilst GABAergic neurons regulate orexinergic neurons to sustain vigilance [28, 35, 43, 44]. As such, the orexin may signal an overall arousal need, defined as a need to counteract actual or potential hazards [45]. In pathologic situations of its reduction, arousal is no longer appropriately coupled to internal and external environment, and sleep-wake instability ensues [45].

Thus, increasingly, the orexinergic system has also been implicated as a key pathophysiological centre for sleep disturbances, pertaining to disorders of hypersomnolence (i.e., narcolepsy type 1, narcolepsy type 2, hypersomnia) [46]. In both animal and human studies, downregulation of the OXR receptors, disrupted OXR signalling, and orexin knockout mice were reported to exhibit narcolepsy, excessive daytime sleepiness, and cataplexy [28, 32, 42, 43, 47–50].

Interestingly, further investigation into orexin receptor polymorphisms have found significant association to specific sleep disturbances in alpha-synucleinopathies [51]. For instance, Rissling and colleagues (2005) conducted an association study analysing the distribution of preprohypocretin polymorphisms in patients with PD with and without sudden onset of sleep [51]. They found significant differences in genotype and allele distribution between patients with PD with, and without, sudden onset of sleep [51]. Particularly, the variant allele T of the preprohypocretin polymorphism was more commonly found in patients with PD with sudden onset of sleep [51]. This suggests a role that genetic factors might play in the pathogenesis of sleep disturbances in alpha-synucleinopathies.

Other clinical studies have also investigated orexin levels using either plasma, spinal CSF or ventricular CSF in alpha-synucleinopathies such as PD [29, 30], MSA [52, 53], and DLB [31, 54], and even in the prodromal stage of alpha-synucleinopathies such as RBD [55, 56]. However, the findings remain inconsistent, with some studies indicating that orexin levels might be lower, elevated, or unaffected in alpha-synucleinopathies [29–31, 54, 57, 58].

To date, available treatments for sleep disorders in alpha-synucleinopathies are only partially effective, partly due to the limited mechanistic insight into their oetiology and hence, we set to systemically review and to obtain a more comprehensive and critical overview of the role of orexin in sleep in patients with alpha-synucleinopathies.

## Methods

### Search Strategies

**Fig. 1:**
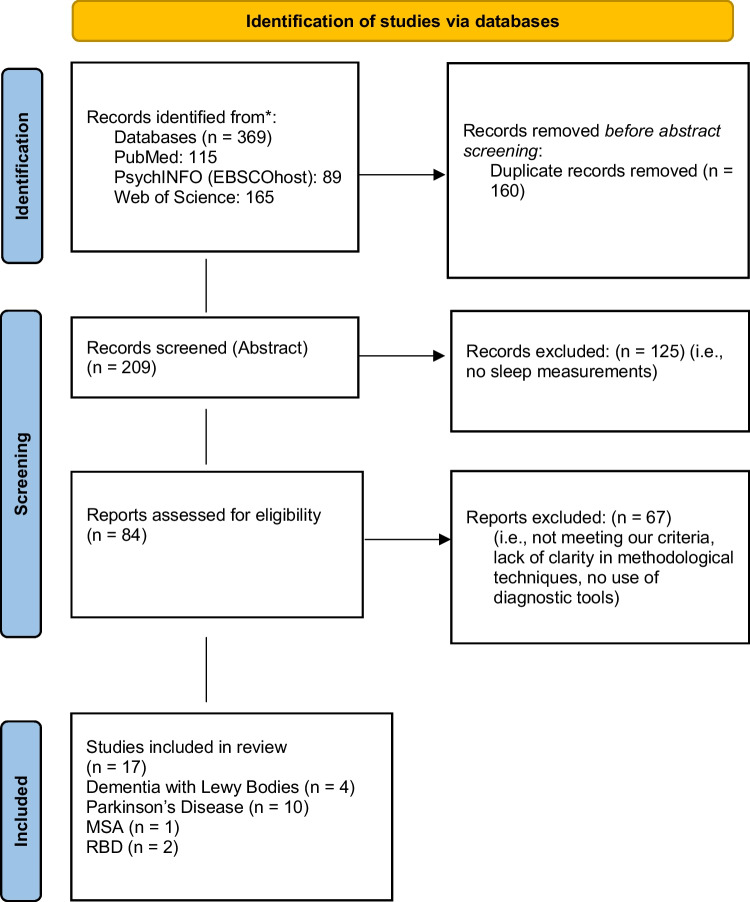
PRISMA 2020 Flow Diagram of study selection process

This systematic review was conducted following the Preferred Reporting Items for Systematic Reviews and Meta-Analyses (PRISMA) guidelines [59] (Fig. 1.). Relevant studies were identified by two reviewers using the electronic databases of PubMed, PsychINFO (EBSCOhost) and Web of Science. The following keywords were used: (Alpha-synucleinopathies OR REM Behaviour Disorder OR Parkinson’s Disease OR Dementia with Lewy Bodies OR Multiple System Atrophy) AND (Orexin) AND (Sleep OR Sleep Disorders OR Sleep Disturbances) (Table 2). Eligible papers were extracted from 2002 until August 2023. The references of the selected articles were also examined to retrieve documents missed by the literature search.

**Table 2:** The search strategy and exclusion/inclusion criteria.

Database	Search Strategy	Limits
PubMed	(Alpha-synucleinopathies) OR (Rem behavior sleep disorder)) OR (Parkinson's Disease)) OR (Dementia with Lewy Bodies)) OR (Multiple System Atrophy)) AND (Orexin)) AND (Sleep OR Sleep Disorders OR Sleep Disturbances)	Year: 2002-2023 Species: Human Age: > 18 Only in English
PsychINFO (EBSCOhost)	(Alpha-synucleinopathies OR REM Behaviour Disorder OR Parkinson’s Disease OR Dementia with Lewy Bodies OR Multiple System Atrophy) AND (Orexin) AND (Sleep OR Sleep Disorders OR Sleep Disturbances)	Year: Inception-2023 Species: Human
Web of Science	(Alpha-synucleinopathies OR REM Behaviour Disorder OR Parkinson’s Disease OR Dementia with Lewy Bodies OR Multiple System Atrophy) AND (Orexin) AND ((Sleep OR Sleep Disorders OR Sleep Disturbances))	Year: Inception-2023 Species: Human

### Inclusion and Exclusion Criteria

Each article was first considered by title and abstract. This systematic review included: 1) original research articles; 2) only papers written in English; 3) observational, descriptive, longitudinal, retrospective, cross-sectional, or cohort studies; 4) studies that investigated orexin, sleep and alpha-synucleinopathies and 5) human studies. Assigned reviewers independently screened each eligible study, and disagreements were resolved through discussion after retrieving full text to determine whether inclusion and exclusion criteria were met (Tables 3 and 4).

**Table 3:** Search criteria

	Exclusion Criteria	Inclusion criteria
Manuscript characteristics	1 Conference abstracts and proceedings, unpublished data, preprints, government publications and reports, dissertations, and theses 2. Animal studies 3. Studies involving under 18s, infants, paediatric. 4. uidelines, statements, and comments 5. General review papers	1. Original research articles 2. Observational, descriptive, longitudinal, retrospective, cross-sectional, cohort studies that included both orexin levels, and sleep measurements in alpha-synucleinopathies 3. Sample was well-described (e.g., number of subjects, recruitment criteria, age mean or age range etc)
Patients’ diagnosis	1. No use of any diagnostic tools	1. Clinical/probable diagnoses of alpha synucleinopathies 2. Parkinson’s Disease (PD): Diagnosis of PD assessed using Unified Parkinson’s Disease Rating Scale (UPDRS) III or the United Kingdom Brain Bank criteria (Hughes et al., 1992) or the International Classification of Diseases, 10th revision (ICD-10), or post-mortem, autopsy confirmation of PD pathology. 3. Dementia with Lewy Bodies: Diagnosis made according to the international consensus criteria (McKeith et al., 1996) or post-mortem, autopsy confirmation of DLB pathology. 4. Multiple System Atrophy (MSA): Diagnosis made according to the Unified Multiple System Atrophy Rating Scale (UM-SARS) Part I and II (Wenning et al., 2004) or post-mortem, autopsy confirmation of MSA pathology. 5. REM Behaviour Disorder (RBD): Diagnosed according to the International Classification of Sleep Disorders (ICSD) criteria (Thorpy, 2012) or polysomnography (PSG)
Sleep quality and Orexin Measurements	1. No measures of sleep quality (i.e., Polysomnography, Multiple Sleep Latency Test (MSLT), Epworth Sleepiness Scale (ESS), Pittsburgh Sleep Quality Index (PSQI) etc.) 2. No measure of orexin levels	1. Objective and subjective measures of sleep quality (e.g. polysomnography, Multiple Sleep Latency Test (MSLT), Epworth Sleepiness Scale (ESS), Pittsburgh Sleep Quality Index (PSQI) etc.) 2. Orexin levels reported (e.g. plasma, ventricular CSF, and spinal CSF)

**Table 4:** The PICOS statement

Component of Question	Example
Patient Population	Alpha-synucleinopathies: Parkinson’s Disease, Dementia with Lewy Bodies, Multiple System Atrophy, REM Behaviour Disorder
Intervention	None
Control	Alpha-synucleinopathies patients and/or healthy controls
Outcomes	Any associations between orexin levels, sleep quality and alpha-synucleinopathies
Study Design	Retrospective, longitudinal, cross-sectional, observational, cohort studies, case-control studies, randomized, controlled trials

### Data Extraction

For each article, two reviewers (KR and SQ) independently extracted the following data: study name and year, the country, type of study, study aim, the subtype of alpha-synucleinopathy, sample size and age of patients, the methods used, main findings and critical evaluation of the study. Subsequently, articles were classified and grouped according to the subtype of alpha-synucleinopathy (i.e., RBD, PD, MSA or DLB).

### Quality Assessment

The quality of studies was evaluated using the Quality Assessment Tool for Quantitative Studies, developed by the Effective Public Health Practice Project (EPHPP; www.ephpp.ca/tool.html) for observational, descriptive, longitudinal, cross-sectional, or cohort studies original research articles [60]. Any disagreements were resolved by discussion with the panel of reviewers, or by consulting with a senior reviewer. For the EPHPP scale, the following criteria were rated for each study on a scale of strong, moderate, or weak: selection bias, study design, blinding, data collection methods, confounders, and withdrawals/attrition (if any). Subsequently, these ratings were compiled to form a global rating: studies were rated as strong if they had no weak ratings, moderate if they had one weak rating, and weak if they received two or more weak ratings.

## Results

### Methodological Quality Assessment Results

Comprehensive tables of the methodological quality assessment of studies using the EPHPP Quality Assessment Tool for Quantitative Studies [60] are displayed in the supplementary section. Quality assessment of studies using the Quality Assessment Tool for Quantitative Studies by EPHPP are presented in Supplementary Table 1. Selection bias was generally rated as moderate as participants in most studies were selected from clinics or hospital settings. Study design was generally rated as moderate as they were observational studies. Studies were rated as weak or strong for confounders. Those rated as weak were because they did not account for potential confounding variables, such as age, disease duration, etc. All studies used valid and reliable measures for sleep (Epworth Sleepiness Scale, Pittsburgh Sleep Quality Index, Multiple Sleep Latency Test etc) and orexin level (radioimmunoassay, ELISA etc). No withdrawals were reported in any of the included studies. No observable association was found between global rating scores and the outcome of the findings.

### Studies characteristics

Our systematic analysis identified 17 studies investigating the association of the orexin pathway and sleep in alpha-synucleinopathies that fulfilled our criteria. These studies were published between the year 2002 and 2022. Among the 17 identified studies, 2 studies included patients with RBD [55, 56], 10 studies with patients with PD [29, 30, 57, 61–67], 4 studies with patients with DLB [31, 54, 58, 68] and 2 studies with patients with MSA [52, 53] (Table 7). It is also worth noting that level of orexin was measured differently in these studies: 12 studies measured orexin levels in spinal CSF [29, 31, 53–57, 61, 63–65, 68], 2 studies used ventricular CSF [62, 66], 2 studies were done on plasma orexin levels only [30, 67] and one study included autopsy findings [58] (Table 5).

**Table 5:** Different measures of orexin in reported studies.

Type of Alpha-synucleinopathies /Location of orexin collection	Spinal CSF	Ventricular CSF	Plasma	Autopsy
REM Behaviour Disorder (RBD)	100% (2/2)	-	-	-
Parkinson’s Disease (PD)	60% (6/10)	20% (2/10)	20% (2/10)	
Dementia with Lewy Bodies (DLB)	75% (3/4)	-	-	25% (1/4)
Multiple System Atrophy (MSA)	100% (1/1)	-	-	-

### Rapid eye movement behaviour disorder (RBD)

Rapid Eye Movement (REM) behaviour disorder (RBD) is a parasomnia characterized by abnormal, violent dream-enacting behaviour, accompanied by an excess of muscle tone (muscle atonia) and/or phasic muscle twitching during REM sleep [69–72]. Most motor events during RBD episodes typically range from simple elementary movements such as laughing, gesturing, or singing to complex, violent behaviours such as punching, kicking, or biting [72–74]. RBD can be categorized into idiopathic RBD (iRBD) when presented with symptoms not ascribable to any other neurological disorders or secondary RBD (sRBD) when associated with other neurological conditions such as narcolepsy type 1 [72, 73, 75]. Importantly, iRBD is increasingly recognised to present an early stage of alpha-synucleinopathy process, such as occur in PD, MSA and DLB [76–79].

In a recent cross-sectional study in China, Yuan and colleagues collected spinal CSF and serum samples from three groups of patients; iRBD, PD with RBD, and PD without RBD, in order to investigate the association between orexin and tumour necrosis factor alpha (TNF-alpha) levels with motor, sleep, and cognitive symptoms [56]. Interestingly, despite abnormal CSF orexin levels (<200 pg/ml) in all the three groups (PD with RBD: 177.69 ± 46.04 pg/ml; PD without RBD: 177.31 ± 29.4 pg/ml and iRBD: 166.23 ± 40.62 pg/ml), orexin levels in the three groups were not reported significantly lower than in the control group [56]. Moreover, no significant difference in CSF orexin levels between the three groups was demonstrated, despite Unified Parkinson's Disease Rating Scale (UPDRS-III) scores being significantly higher in the PD with RBD and PD without RBD groups and REM sleep behavior disorder questionnaire (RBDQ-HK) scores significantly higher in RBD, perhaps suggesting that orexin levels may not be a sensitive marker for motor severity and sleep behaviours [56].

However, these findings are somewhat inconsistent with an earlier study that measured CSF orexin levels in five iRBD patients and control group (consisting of narcolepsy and multiple sclerosis patients) and reported slightly higher orexin levels between 250 and 454 pg/ml (mean 369.2 ± 43.91 pg/ml) in iRBD group [55]. In this study, the Epworth Sleepiness Score (ESS) was reported normal at 5.3 (scored out of 24 points) in iRBD patients, which may suggest an underlying compensatory mechanism at play [55]. Several methodological factors could explain these contrasting findings. Firstly, the small sample sizes of both studies increase likelihood of speculative findings [55]. Secondly, in study by Yuan and colleagues, it is not clear whether RBD appeared before or after motor symptoms, and thus, set of different neuropathologic trajectories may be at play here [56]. Additionally, neither study made attempt at classification according to patients’ respective medication status, and as such it may not be reflective of the observable symptomatic behaviours (i.e., any sleep disturbances). Consequently, results of sleep scales such as ESS and RBDQ may be insufficiently sensitive to reflect the actual clinical status. Finally, perhaps unsurprisingly, neither study demonstrated an association between sleep, or disease severity measures, and orexin levels.

### Parkinson’s Disease

Parkinson’s disease (PD) is the second most common neurodegenerative disorder associated with the presence of intraneuronal Lewy bodies and Lewy neurites, loss of dopaminergic neurons in the substantia nigra, aberrant intracellular alpha-synuclein protein aggregations and neuroinflammation [80, 81]. Consequently, patients with PD exhibit a diverse range of motor and non-motor symptoms including resting tremor, bradykinesia, autonomic dysfunction, cognitive deficits, depression, and postural instability [82, 83]. Of non-motor symptoms, they also frequently display a range of general and more distinct sleep disturbances, including insomnia, sleep attacks (not unlike those in narcolepsy) [27], excessive daytime sleepiness, as well as parasomnias, such as REM Behaviour Disorder [84–94].

As a result of the high prevalence of sleep disturbances in patients with PD, there is a growing interest in orexin as fluctuations in CSF orexin and degeneration of orexinergic neurons have been associated with the development of sleep disorders that mimic early stages of alpha-synucleinopathy in other groups of patients (e.g. secondary RBD in narcolepsy) [29, 30, 57, 61–65, 95].

Several studies failed to demonstrate significant changes in orexin levels in PD patients [63, 64]. For example, Asai and colleagues measured CSF orexin levels in three groups of patient with PD; those without sleep disturbances, those with excessive daytime somnolence, and patients with sleep attacks and found no significant differences in orexin levels between the three groups, although orexin levels negatively correlated with disease duration [63]. Additionally, Compta and colleagues also measured CSF orexin-A levels in patient with PD with dementia, and without dementia [64]. Interestingly, despite higher ESS scores in both groups than in controls, there was no difference in CSF orexin-A levels between the three groups [64].

Orexin has been argued to play a neuroprotective and immune-regulatory role in the central nervous system, which may be affected in later stages of alpha-synucleinopathy process, when alpha-synuclein may act to promote the degradation of OX1R through lysosomal and proteasomal pathways [30, 96]. In that light, another study that reported reduced orexin levels in hyper-somnolent PD patients, as compared to controls, is of interest [57]. Here, glial fibrillary acidic protein (GFAP) levels, suggestive of neuroinflammation, were reported as significantly higher in patients with hypersomnolence and narcolepsy, but not in patient with PD without hypersomnolence [57].

In past, several studies also reported lower orexin CSF levels in patients with PD. For instance, in a retrospective study conducted in China that investigated association between CSF orexin levels, sleep, and clinical characteristics the significantly lower levels of CSF orexin in patients with PD (median = 272 pg/mL), as compared to controls (median = 352.2 pg/mL), were demonstrated [29]. In keeping, in another retrospective, case-control, post-mortem study of patients with PD, a reduced number of orexinergic neurons, and lower CSF and prefrontal orexin levels were similarly reported [95]. Moreover, lower CSF orexin levels, correlated with PD disease severity, were also reported [66]. Notably, Wienecke and colleagues [61] measured repeated CSF orexin levels in two patients with PD and reported a marked decrease of orexin concentrations over time, suggesting a progressive loss of orexin as disease advances [61].

Notwithstanding this, an uneven polymorphic picture emerges from studies where the association of orexin levels to specific sleep characteristics was investigated. For example, Ogawa and colleagues report no significant correlation between CSF orexin levels and PD clinical characteristics, including excessive daytime somnolence scores, presence of RBD, or use of dopamine agonists [29]. Moreover, this lack of association remained for the duration of a follow up [29]. In striking contrast, Wienecke and colleagues have demonstrated a strong link between CSF orexin levels and the mean sleep latencies on MSLT [61].

In regards to links between orexin and sleep macrostructure, Bridoux and colleagues have reported a significant positive correlation between orexin-A levels and the percentage of REM epochs without atonia, but they failed to demonstrate any correlation with total sleep time, REM duration or REM latency [62]. Strikingly, however, in this study, patients with loss of REM atonia had higher median orexin levels than the four patients with REM sleep atonia (235 pg/mL versus 94 pg/mL) [62].

Whilst the pathomechanisms behind these diverse findings remain unclear, a potential insight could be gleamed from the prospective study by Huang and colleagues [30]. They report an initial increase in orexin-A levels in early-stage and medium-stage PD, with decreased levels then demonstrated in advanced stage of PD [30]. These findings suggests that orexin levels may reflect the dynamic changes occurring in disease severity and disease duration.

Other methodological and physiological factors could also potentially account for these polymorphic findings (see Table 6). Firstly, sampling of orexin from different areas and times may explain differences in orexin level; e.g. orexin levels fluctuate over the day due to circadian rhythmicity [97]. Also, orexin level in spinal CSF is reportedly reduced in acute neurological disorders, whilst ventricular CSF orexin correlated with prefrontal cortex orexin concentration [65]. Secondly, in a similar vein, reduction in CSF orexin level depends heavily on the level and progression of degeneration of orexin neurons in the lateral hypothalamus [98]. For instance, in a study investigating the relationship between CSF orexin levels and orexin neuronal loss, Gerashchenko and colleagues reported that maximal reduction of CSF orexin level was only observed on day 60 without further decline or recovery, following a neurotoxin-induced lateral hypothalamic injury [98]. This suggests CSF orexin level may not be a very sensitive indicator of loss of orexinergic neurons in the early stage of PD as remaining orexinergic neurons engage in a compensatory mechanism to counteract and increase CSF orexin levels.

**Table 6:** Key Summary Information of all Identified Studies

Study	Type	Country	Design	Location of orexin collection		Time of Orexin Collection (if stated)	Included patients on Medications (if stated)	Patient Group			Control Group			p- value
								Sample Size	Age (Years)	Orexin-A level (pg/ml)	Sample Size	Age (Years)	Orexin-A level (pg/ml)	
Anderson et al. (2010)	RBD	UK	Retrospective, cross-sectional	Spinal CSF		-	-	5	61.6	369.2 ± 43.91	11	Median = 29	Median = 279	-
Yuan et al. (2022)	RBD	China	Retrospective, cross-sectional	Spinal CSF		-	-	iRBD = 7 PD+RBD = 9 PD-RBD = 4	63.3	iRBD = 166.23 ± 40.62 PD+RBD = 177.69 ± 46.04 PD-RBD = 177.31 ± 29.4	10	63.3	218.83 ± 43.46	0.224
Wienecke et al., (2012)	PD	Switzerland	Prospective, case-controlled	Spinal CSF		-	-	aPD = 7 ePD = 7	aPD = 64 ePD = 59	aPD = 487 ± 57 ePD = 525 ± 63	10	63	-	-
Ogawa et al., (2022)	PD	Japan	Cross-sectional, longitudinal	Spinal CSF		In the morning after overnight fasting	Dopamine Agonists	58	69	221.Cro7 – 334.5	20	72	296.2 – 399.5	0.007
Drout et al., (2003)	PD	France	Cross-sectional	Ventricular CSF		Between 10 am and 12 am	Levodopa agonists: Ropinirole, pergolide, amantadine, entacapone, trihexyphenidyl, bromocriptine, selegiline. Psychotropic medications: Benzodiazepines, paroxetine, mianserine, amitriptyline, sertraline, maprotiline, meprobamate, buspirone, clomipramine, zolpidem, baclofen, citalopram	19	-	57 ± 14	5	-	199 ± 34	0.002
Afdal et al., (2021)	PD	Indonesia	Cross-sectional	Plasma CSF		-	-	60	63.42	PD with sleep disorders = 81.817 ± 22.770 PD without sleep disorders = 255.416 ± 226.590	-	-	-	-
Huang et al., (2021)	PD	China	Prospective, case-controlled	Plasma CSF		-	L-Dopa and Dopamine receptor agonists	121	64	1.13 ± 0.27 ng/mL	117	62	0.94 ± 0.26 ng/mL	< .001
Takahashi et al., (2015)	PD	Japan	Cross-sectional	Spinal CSF		-	L-Dopa	HS = 5 wHS = 8	-	See table	9		See Table	
Bridoux et al., (2013)	PD	France	Cross-sectional	Ventricular CSF		Between 10 am and 12 am	-	8	-	See table	-	-	-	-
Asai et al., (2009)	PD	Japan	Cross-sectional	Spinal CSF		Before breakfast	-	25	66.2	285.2 ± 77.4	12	37	167 – 419	-
Compta et al., (2009)	PD	Spain	Cross-sectional	Spinal CSF		Before morning dose of anti-parkinsonian medications	L-Dopa	PDD = 20 PDND = 21	PDD = 72.5 PDND = 68.8	PDD = 309.94 ± 65.95 PDND = 300.99 ± 58.68	22	70.4	321.15 ± 47.15	0.67
Yasui et al., (2006)	PD	Japan	Cross-sectional	Spinal CSF		-	L-Dopa and other anti-parkinsonian drugs	PD = 62 DLB = 13	PD = 69.5 DLB = 75.7	PD = 302 ± 38 DLB = 297 ± 48	-	-	-	-
Inagawa et al., (2021)	DLB	Japan	Cross-sectional	Spinal CSF		-	-	19	81.1	271.6 ± 114.5	25	71.4	301 ± 64	>.05
Lessig et al., (2010)	DLB	USA	Cross-sectional	Autopsy		-	-	21	79.5	See table	-	-	-	-
Baumann et al., (2004)	DLB	Switzerland	Cross-sectional, case-controlled	Spinal CSF		Between 9 am and 6 pm	L-Dopa and Cholinesterase Inhibitor	10	72.4	521	20	44	497	>.05
Trotti et al., (2021)	DLB	USA	Cross-sectional	Spinal CSF		No control for time of collection	-	20	64.9	240.3 ± 60.2	25	69.6	248.1 ± 53.3	0.79
Abdo et al., (2008)	MSA	The Netherlands	Retrospective	Spinal CSF		-	-	12	60	343	11	52	339	0.34
Martinez et al., (2007)	MSA	Spain	Cross-sectional	Spinal CSF		Between 9 am and 1 pm	L-Dopa	15	60.6	245 ± 35	6	54.3	269 ± 22	>.05
Study	Type	Country	Design	Mean ESS (if any)	Location of orexin collection	Time of Orexin Collection (if any)	Included patients on Medications (if stated)	Patient Group			Control Group			p- value
								Sample Size	Age (Years)	Orexin-A level (pg/ml) (mean ± SD)	Sample Size	Age (Years)	Orexin-A level (pg/ml)	
Anderson et al. (2010) [55]	RBD	UK	Retrospective, cross-sectional	RBD = 5.3	Spinal CSF	None	Antihypertensive enalapril, aspirin, metformin	5	61.6	369.2 ± 43.91	11	Median = 29	Median = 279	>.05
Yuan et al. (2022) [56]	RBD	China	Retrospective, cross-sectional	PD+ RBD = 4.5 PD – RBD = 5 iRBD = 4.88	Spinal CSF	None	Not stated	iRBD = 7 PD+RBD = 9 PD-RBD = 4	63.3	iRBD = 166.23 ± 40.62 PD+RBD = 177.69 ± 46.04 PD-RBD = 177.31 ± 29.4	10	63.3	218.83 ± 43.46	0.224
Wienecke et al. (2012) [61]	PD	Switzerland	Prospective, case-controlled	aPD = 10 ePD = 10 Controls = 7	Spinal CSF	None	Levodopa, antidepressants	aPD = 7 ePD = 7	aPD = 64 ePD = 59	aPD = 487 ± 57 ePD = 525 ± 63	Narcolepsy with cataplexy (NC) = 10 Controls = 10	63	NC = 14 ± 21	aPD and ePD vs. NC: <.001
Ogawa et al. (2022) [29]	PD	Japan	Cross-sectional, longitudinal	PD = 8	Spinal CSF	In the morning after overnight fasting	Dopamine Agonists	58	69	221.7 – 334.5	20	72	296.2 – 399.5	0.007
Drouot et al. (2003) [66]	PD	France	Cross-sectional	PD = 11	Ventricular CSF	Between 10 am and 12 am	Dopamine agonists (ropinirole, pergolide, apomorphine, bromocriptine) and other antiparkinson drugs (entacapone, amantadine, anticholinergics, and selegiline.	19	NA	57 ± 14	5	NA	199 ± 34	0.002
Afdal et al. (2021) [67]	PD	Indonesia	Cross-sectional	Not stated	Plasma	None	Excluded patients who are taking drugs that have a sedative effect (e.g., antihistamines, muscle relaxants)	PD with sleep disorders = 30	64.43 ± 6.73	81.82 ± 22.77	PD without sleep disorders = 30	62.4 ± 9.76	255.42 ± 226.59	.000
Huang et al. (2021) [30]	PD	China	Prospective, case-controlled	PD = 9	Plasma	Between 07:30 and 8:30 am, after overnight fasting and before breakfast	L-Dopa and Dopamine receptor agonists	121	64	1.13 ± 0.27 ng/mL	117	62	0.94 ± 0.26 ng/mL	< .001
Takahashi et al. (2015) [57]	PD	Japan	Cross-sectional	NA	Spinal CSF	None	L-Dopa, amantadine, cabergoline	HS = 5 wHS = 8	NA	See Table 7.	9	NA	NA	NA
Bridoux et al. (2013) [62]	PD	France	Cross-sectional	NA	Ventricular CSF	Between 10 am and 12 am	Excluded patients with sleep-altering medications	8	58.3	187.9	NA	NA	NA	NA
Asai et al. (2009) [63]	PD	Japan	Cross-sectional	PD (Total) = 8.4	Spinal CSF	Before breakfast	L-dopa, pramipexole, pergolide	25	66.2	285.2 ± 77.4	12	37	167 – 419	NA
Compta et al. (2009) [64]	PD	Spain	Cross-sectional	PDD = 13.3 PDND = 10.34 ± 5.6 Controls = 5.32	Spinal CSF	Before morning dose of anti-parkinsonian medications	L-dopa, dopamine agonists	PDD = 20 PDND = 21	PDD = 72.5 PDND = 68.8	PDD = 309.94 ± 65.95 PDND = 300.99 ± 58.68	22	70.4	321.15 ± 47.15	0.67
Yasui et al. (2006) [65]	PD	Japan	Cross-sectional	NA	Spinal CSF	Under 12-16 hours fasting conditions	L-Dopa and other anti-parkinsonian drugs	PD = 62 DLB = 13	PD = 69.5 DLB = 75.7 PSP = 72 CBD = 71.4	PD = 302 ± 38 DLB = 297 ± 48	NA	NA	NA	NA
Inagawa et al. (2021) [31]	DLB	Japan	Cross-sectional	NA	Spinal CSF	None	Excluded patients who took medications that can affect sleep disturbance profile, cognitive function, and CSF orexin levels (i.e., cholinesterase inhibitors, antidepressants, and hypnotics)	19	81.1	271.6 ± 114.5	25	71.4	301 ± 64	>.05
Lessig et al. (2010) [58]	DLB	USA	Cross-sectional (Autopsy)	NA	Autopsy	NA	-	21	79.5	See Table 7.	3	85.7	-	-
Baumann et al. (2004) [68]	DLB	Switzerland	Cross-sectional, case-controlled	DLB (9 patients, 1 unavailable) = 12.7	Spinal CSF	Between 9 am and 5 pm	L-Dopa and Cholinesterase Inhibitor	10	72.4	521	20	44	497	>.05
Trotti et al. (2021) [54]	DLB	USA	Cross-sectional	DLB = 9.8 Controls = 4.8	Spinal CSF	Without overnight fasting	Cholinesterase inhibitors	20	64.9	240.3 ± 60.2	25	69.6	248.1 ± 53.3	0.79
Martinez-Rodriguez et al. (2007) [53]	MSA	Spain	Cross-sectional	MSA = 5.7	Spinal CSF	Between 9 am and 1 pm	L-Dopa	15	60.6	245 ± 35	6	54.3	269 ± 22	>.05

Abbreviations: *CSF* Cerebrospinal Fluid, *DLB* Dementia with Lewy Bodies, *EMG* Electromyography, *iRBD* idiopathic RBD, *MSA* Multiple System Atrophy, *NA *Not Available, *PD* Parkinson’s Disease, *PDD* Parkinson’s Disease with Dementia, *PDND* Parkinson’s Disease without Dementia, *RBD* REM Sleep Behaviour Disorder, *PSP* Progressive Supranuclear Palsy

**Table 7:** List of studies’ information, methods, main findings, and critical evaluation of orexin and sleep disturbances in alpha-synucleinopathies (RBD, PD, DLB and MSA) from 2002 to 2022

Author/Year Country Type of Study	Subtype	Sample size (age at time of study unless stated otherwise)	Methods	Main findings	Critical Evaluation
REM Behaviour Sleep Disorder (RBD)					
Anderson et al. (2010) Cambridge, UK Retrospective, cross-sectional study	iRBD	iRBD: N = 5 (mean age = 61.6) M = 4 F = 1 Controls: N = 11 (median age = 29) (10 with multiple sclerosis, 1 with narcolepsy and cataplexy)	1. Detailed neurological history and examination 2. Hypocretin-1 analysis: CSF fluid extracted and measured by a competitive radioimmunoprecipitation assay.	1. Orexin levels in iRBD patients are slightly higher but not significantly different from controls. More specifically, hypocretin levels were between 254 and 450 pg/ml and therefore within the normal range of >100pg/ml. 2. The mean ESS score was normal at 5.3	1. Controls were younger individuals (ranging from 4 to 69, median – 29 years old), which is significantly younger than the iRBD patients. 2. Age, gender, and sampling time could be cofounders. 3. Small sample size
Yuan et al. (2022) Beijing, China Retrospective, cross-sectional study	iRBD PD	iRBD: N = 52 (63.33 ± 8.88) PD + RBD: N = 38 (64.35 ± 9.83) PD – RBD: N = 55 (59.94 ± 9.17)	Demographics and Clinical Assessments: 1. Motor: UPDRS-III, H&Y 2. Sleep: PSQI, ESS, RBDQ-HK 3. Cognition: MoCA 4. Mood: HAMA, HAMD Video-PSG CSF and Serum Samples: 1. CSF was collected via lumbar puncture. 2. Level of orexin was measured by chemiluminescent immunoassay. 3. TNF-A was evaluated using the IMMULITE/IMMULITE 1000 TNF-A kits.	CSF analyses: 1. There was no significant difference in CSF TNF-a levels among the three groups (4.2 ± 0.37 vs. 4.47 ± 0.58 vs. 6.28 ± 2.2 pg/ml, p = 0.368) 2. CSF orexin levels were abnormal (<200 pg/ml) in all PD+RBD, PD-RBD and iRBD patients (177.69 ± 46.04, 177.31 ± 29.4, 166.23 ± 40.62 pg/ml), although the orexin levels of the three groups were not significantly lower than the control group. 3. There was no significant correlation between the levels of TNF-a and orexin (p = 0.647) Clinical Assessments: 1. UPDRS-III scores were significantly higher in the PD+RBD group and PD–RBD groups than in the iRBD group. 2. There is no statistically significant difference in H&Y stages, PSQI, or ESS scores between the PD+RBD and PD-RBD groups. However, RBDQ-HK scores in the iRBD group were significantly higher than other groups. 3. Sleep architecture did not differ significantly among the three groups, except for a higher proportion of REM sleep in iRBD patients, as compared to the PD-RBD group.	1. In patients who do not complain of abnormal behaviours in their sleep, the object of inquiry about the PSQI and RBDQ-HK should be the family members or caregivers of the patient, rather than the patients themselves. 2. This study did not further divide patients with or without therapeutic medication into subgroups. 3. PD with RBD patients were not further classified into subgroups depending on whether RBD appeared before or after motor symptoms of PD, so the time node at which RBD first appears may be related to the severity of neuroinflammatory reactions in the central and peripheral nervous system. 4. Small sample size
Parkinson’s Disease					
Wienecke et al. (2012) Zurich, Switzerland Prospective, controlled study	PD	N = 30 Advanced PD (aPD) = 10 Early & Treatment-naïve PD (ePD) = 10 Controls = 10 Additional retrospective data (Narcolepsy with cataplexy): N = 10	Demographics and Clinical Assessments: 1. Clinical interview with patient (regarding sleep paralysis, hallucinations and RBD) 2. ESS PSG MSLT Spinal CSF-Orexin Measurement	PSG and MSLT: 1. Compared to controls, aPD had lower sleep efficiency (p = 0.02), less REM sleep (p = 0.01) and less deep sleep (p = 0.01), whilst ePD and controls had similar sleep architecture in all PSG parameters 2. In PD patients, mean sleep latencies on MSLT were correlated negatively with disease duration (p < 0.002) CSF Analyses: 1. In PD patients, CSF orexin levels correlated with mean sleep latencies on MSLT (p = 0.02). Conversely, there was no correlation between CSF orexin and disease duration, age, LEDD, or dopamine agonist dose. 2. Repeated CSF orexin measurements in 2 aPD patients (intra-individual recording) revealed a marked decrease of concentrations over time, indicating a progressive loss of orexin over the course of PD, suggesting that dopamine and hypocretin deficiency differentially affect sleep and wakefulness in PD.	1. Small sample size 2. Age and gender not matched between NC and PD patients. 3. As controls with sleep apnea was excluded, the slightly higher apnoea severity in aPD patients might have affected sleep fragmentation and structure.
Ogawa et al. (2022) Osaka, Japan Cross-sectional, longitudinal study	PD	PD: N = 58 (69) Controls: N = 20 (72)	Demographics and Clinical Assessments: 1. Clinical data: age, gender, history, disease duration, education, medication status (LEDD) 2. Sleep: ESS, RBDSQ 3. Disease severity: H&Y, UPDRS, PDQ-39 4. Cognition: MMSE, FAB 5. Non-motor symptoms: Olfactory Identification score, SCOPA-AUT, GDS-15, AS CSF Collection and Analyses: 1. CSF was collected by lumbar puncture in the morning after overnight fasting, keeping the usual medications. 2. CSF orexin levels were measured using a radioimmunoassay kit.	1. CSF orexin levels in PD patients (median = 272.0 [interquartile range = 221.7 – 334.5] pg/mL) were lower than those in controls (352.2 [296.2 – 399.5] pg/mL, p = 0.007). 2. There are no significant differences in CSF orexin levels according to EDS, RBD, or the use of dopamine agonists. 3. No significant correlation was observed between CSF orexin levels and clinical characteristics by multiple linear regression analysis. 4. Additionally, the longitudinal changes in orexin levels were also not correlated with clinical characteristics, suggesting the limited efficacy of CSF orexin levels as a biomarker for PD, and that sleep disturbances may also be affected by dysfunction of the nervous system other than orexin or by dopaminergic treatments in PD.	1. Small sample size 2. EDS and RBD were evaluated by only a subjective questionnaire, so they may not reflect the objective sleep period and rhythms. 3. This study included only a few patients with advanced PD, and the intervals from the initial examinations were within three years. 4. Retrospective nature of the study 5. This study recruited patients who were not diagnosed with neurodegenerative disease, including those with normal pressure hydrocephalus, as controls – these patients potentially differ from the healthy elderly. 6. All patients included in this study had optimal medication during examination, therefore, the impact of dopamine replacement therapy could not be ruled out.
Drout et al., (2003) Créteil, France Cross-sectional study	PD	PD: N = 19 (61 ± 2) Controls: N = 5 (47 ± 9)	Demographics and Clinical Assessments: 1. Clinical data: age, gender, history, disease duration, medication status (LEDD) 2. Sleep: ESS 3. Disease severity: UPDRS CSF Collection and Analyses: 1. Ventricular CSF 2. Orexin A (hypocretin 1) was measured in crude CSF using a commercially available radioimmunoassay kit.	1. Lower ventricular CSF orexin in PD patients than in controls (p = 0.01), and orexin levels negatively correlated with diseases severity (p = 0.03). 2. PD patients reported more sleepiness in ESS than controls: Decreased orexin production may contribute to the development of EDS in late-stage PD and may be an intrinsic factor in the pathophysiology of EDS.	1. Small sample size
Afdal et al., (2021) Padang, Indonesia Cross-sectional study	PD	PD with sleep disordes: N = 30 PD without sleep disorders: N = 30	Demographics and Clinical Assessments: 1. Clinical data: age, gender, history, disease duration, medication status (LEDD) 2. Sleep: ESS 3. Disease severity: H&Y CSF Collection and Analyses: 1. Orexin levels were measured using the ELISA method.	1. There was a significant difference in lower plasma hypocretin levels in PD with sleep disorders (81.817 ± 22.77) and in the group without sleep disorders (255.42 ± 226.59) (p = .000). 2. There was no statistical difference in clinical degree, age, duration of illness between the PD group with sleep disorders and the group without sleep disorders (p > 0.05).	1. Small sample size
Huang et al. (2021) Zhengzhou, China Prospective, case-controlled study	PD	PD: N = 121 (mean age = 64) Controls: N = 117 (mean age = 62)	Demographics and Clinical Assessments: 1. Clinical data: age, gender, history, disease duration, education, medication status (LEDD) 2. Disease severity: UPDRS-III, H&Y 3. Sleep: PSQI, RBDQ-HK, ESS, RBD, EDS 4. Cognition: MMSE 5. Mood and Neuropsychiatric: HAMD-17, HAMA-14, FSS, AS 6.Non-motor symptoms: NMSS, AHRS Blood sampling and Plasma Orexin-A levels analysis: 1. Peripheral blood sample were obtained from each subject. 2. Plasma orexin-A levels were measured using an enzyme-linked immunosorbent assay (ELISA) kit.	1. Plasma orexin-A levels were significantly higher in PD than controls. More particularly, orexin-A levels were elevated in early-stage and medium-stage PD than controls (p < 0.001; p = 0.002, respectively) but levels decreased in advanced stage PD (p = 0.014). 2. Orexin-A levels were positively correlated with the severity of insomnia and RBD, and levels of orexin-A in PD with insomnia, and RBD were increased. 3. Orexin-A levels were negatively correlated with UPDRS-III scores, disease duration, and dopamine receptor agonist doses, and were positively correlated with the PSQI, RBDSQ, HAMA, MMSE, and NMSS item 22-24 scores.	1. Controversial idea of whether plasma orexin-A levels reflect central orexin-A activity. 2. Non-motor symptoms were assessed using scales, which may be influenced by subjective factors. 3. This study is a cross-sectional study; Longitudinal studies are needed to account for possibilities of confounders like age, sex, medications, and disease trajectory
Takahashi et al. (2015) Akita, Japan Cross-sectional study	PD Narcolepsy	PD: N (with HS) = 5 N (without HS) = 8 Narcolepsy: N = 8 Controls: N = 9	Demographics and Clinical Assessments: 1. Clinical history 2. Sleep: MSLT, ESS CSF Collection and Analyses: 1. Levels of orexin, GFAP and S100B in CSF were measured. 2. Orexin level was measured by radioimmunoassay and levels of GFAP and S100B were measured by GFAP ELISA Kit and a S100B ELISA Kit	1. The levels of CSF orexin in PD patients with HS tended to be lower compared to those in control group, although this difference was not statistically significant. 2. Compared with those of the control group, GFAP levels were significantly higher in the group of PD with HS and narcolepsy, but not PD without HS. 3. No groups showed a significant difference in S100B levels. 4. In the whole subjects with PD, narcolepsy, and controls, orexin levels were inversely correlated with GFAP. Hence, this suggests that increased GFAP may indicate orexin deficiency both in narcolepsy and PD with HS.	1. Complete information on disease state of PD, sleep-related symptoms (sleep apnea, RBD, restless legs syndrome) and sleep examination were not available, and degree of HS could not be examined in detail
Bridoux et al. (2013) Chenevier, France Cross-sectional study	PD	PD: N = 8 M = 4 F = 4	Demographics and Clinical Assessments: 1. Clinical history 2. Disease severity: UPDRS-III PSG CSF Collection and Analyses: 1. Ventricular CSF were obtained via ventriculography 2. Orexin-A (hypocretin-1) was measured in crude CSF using a commercially available radioimmunoassay kit.	1. Orexin-A levels showed a significant positive correlation with the percentage of REM epochs without atonia (p = 0.07). 2. Orexin-A levels did not correlate with total sleep time (p = 0.23), REM duration (p = 0.45), or REM latency (p = 0.66) 3. Four patients included had RSWA, and these four patients had higher median (interquartile range) orexin-A levels than the four patients with REM sleep atonia (235 pg/mL [215-345] vs 94 pg/mL [44-147]; p = 0.036)	1. Small sample size 2. No use of controls 3. This study excluded patients taking sleep-altering medications and EMG activities concurrent with respiratory arousals
Asai et al. (2009) Nara, Japan Cross-sectional study	PD	PD: N = 25 (Age at onset = 66.2 ± 8.6) M = 14 F = 11 Narcolepsy with cataplexy: N = 56 (28.5 ± 20.6) Controls: N = 12 (37 ± 15)	Demographics and Clinical Assessments: 1. Clinical history 2. Disease severity: H&Y 3. Sleep: ESS, Sleep attack (diagnosed according to Homann’s criteria) CSF levels collection and analyses: 1. Spinal CSF were obtained by lumbar puncture 2. Orexin in crude CSF was measured in duplicate using a commercially available radioimmunoassay kit.	1. CSF-orexin levels did not significant differ between PD without sleep disturbances (302.3 ± 71.1 pg/ml), PD with EDS (286.1 ± 47.6), and PD with SAs (232.4 ± 12.4) but were decreased in narcolepsy patients (94.8 ± 109.6) 2. Orexin levels negatively correlated with disease duration (p < 0.05) 3. Orexin levels did not correlate with ESS (p = 0.98, r = -0.67), suggesting no relationship between orexin levels and EDS 4. Replacing drugs with D3 stimulatory activity with Pergolide significantly increase CSF-orexin levels with baseline (p = 0.034), which is associated with complete resolution of SAs	1. Use of spinal CSF for measurement of orexin is not as sensitive as ventricular CSF. 2. Small sample size
Compta et al. (2009) Barcelona, Spain Cross-sectional study	PD	PDD (PD with dementia): N = 20 (72.5 ± 7.14) PDND (PD without dementia): N = 21 (68.8 ± 6.9) Controls: N = 22 (70.4 ± 9)	Demographics and Clinical Assessments: 1. Clinical history and review 2. Cognition: MMSE 3. Sleep: ESS 4. Disease Severity: UPDRS-III, H&Y 5. Medications CSF levels collection and analyses: 1. Spinal CSF were obtained by lumbar puncture 2. CSF hypocretin-1 levels were determined with commercially available direct radio-immunoassay kit. PSG MSLT	1. There was no difference in CSF Orexin-A between the three groups (p = 0.67). 2. Altered NREM sleep and/or slow dominant occipital frequency was more frequent in PDD than PDND (p = 0.029). 3. ESS scores were higher in both PD groups than in controls, and EDS defined as ESS > 10 was more frequent in PDD group.	1. Selection bias due to the convenient sampling 2. Auto-administered scale may develop inaccuracies. 3. Sleep studies were only given to a subset of PD patients and none of the controls, leading to underpowered analysis for this part.
Yasui et al. (2006) Yonago, Japan Cross-sectional study	PD DLB PSP CBD	PD: N = 62 (69.5 ± 9.7) DLB: N = 13 (75.7 ± 6.3) PSP: N = 16 (72.0 ± 5.9) CBD: N = 7 (71.4 ± 8.1)	Demographics and Clinical Assessments: 1. Clinical history and review 2. Sleep: EDS CSF levels collection and analyses: 1. Spinal CSF were obtained by lumbar puncture 2. CSF Orexin A levels were measured using radioimmunoassay.	1. Levels of CSF orexin (mean ± SD pg/ml) were 302 ± 38 in PD, 297 ± 48 in DLB, 258 ± 37 in PSP, 246 ± 90 in CBD. 2. The occurrence of low orexin levels (<110 pg/ml) was rare in both PD and DLB, and orexin levels were significantly lower in the PSP and CBD groups compared to PD (PSP: p < 0.001, CBD: p < 0.05). 3. Orexin levels were inversely correlated with duration of morbidity in PSP but not in the other conditions, suggesting that loss of orexin neurons or impaired orexin neurotransmission might exist as a part of the neurodegeneration associated with advanced PSP with long duration of morbidity.	1. Small sample size 2. No controls used
Dementia with Lewy Bodies (DLB)					
Inagawa et al. (2021) Tokyo, Japan Cross-sectional study	DLB RBD	DLB: N = 19 (81.1 ± 3.1) M = 10 F = 9 AD: N = 22 (73.9 ± 8.0) Controls: N = 25 (71.4 ± 11.2)	Demographics and Clinical Assessments: 1. Clinical history and review 2. Cognition: MMSE, MoCA 3. Sleep: NPI, RBDQ-J 4. Caregiver distress: NICDS CSF levels collection and analyses: 1. Spinal CSF were obtained by lumbar puncture 2. CSF Orexin A levels were measured using radioimmunoassay.	1. There were no significant differences in mean ± standard deviation CSF Orexin A levels between DLB patients (271.6 ± 114.5 pg/mL) and controls (301 ± 64 pg/mL) or AD patients. 2. No significant correlation was observed between cognitive function (i.e., MMSE and MoCA) and CSF Orexin A levels in DLB patients. 3. However, in DLB patients, there was a significant positive correlation between the severity of sleep disturbance and CSF Orexin A level.	1. Small sample size
Lessig et al. (2010) La Jolla, California Cross-sectional study	DLB AD	DLB: N = 21 (79.5 ± 7.7) AD: N = 19 (82.5 ± 9.0) Controls: N = 3 (85.7 ± 4.6)	Case selection and neuropathological evaluation: 1. Autopsy material was obtained from patients who received neurological and psychometric testing 12 months before death. 2. A sleep evaluation was obtained including sleep difficulty, frequency, and leg movements during sleep. 3. Autopsy procedures and neuropathological diagnosis Immunohistochemistry Immunoblot Analysis	1. Whilst limited hypocretin alterations were observed in AD, this study demonstrated reduced neocortical hypocretin-immunoreactivity in DLB patients, correlating with hypersomnolence and alpha-synuclein levels. This result suggests the involvement of hypocretin in sleep disorders in DLB.	1. Small sample size 2. Autopsy makes it difficult to link any specific sleep parameters to orexin
Baumann et al. (2004) Zurich, Switzerland Cross-sectional, case-controlled study	DLB with EDS	DLB: N = 10 (72.4) AD: N = 7 (72) Controls: N = 20 (44)	Demographics and Clinical Assessments: 1. Clinical history and review 2. Subjective estimation of severity of excessive daytime sleepiness (EDS) MLST PSG CSF levels collection and analyses: 1. CSF were obtained via lumbar puncture. 2. Measurements of hypocretin-1 was performed by radioimmunoassays (RIA)	1. CSF orexin levels were found to be in the normal range (mean 521 pg/ml, range 382-667) when compared to controls (mean 497 pg/ml, range 350-603) and AD (mean 474pg/ml, range 333-564). 2. In DLB, excessive daytime sleepiness, hallucinations and RBD may occur in the absence of a detectable hypocretin deficiency	1. Small sample size
Trotti et al. (2021) Atlanta, USA Cross-sectional study	AD FTD DLB	AD: N = 60 (65.5 ± 7.7) FTD: N = 21 (66.3 ± 9.2) DLB: N = 20 (64.9 ± 9.8) Controls: N = 25 (69.6 ± 9.2)	Demographics and Clinical Assessments: 1. Clinical history and review 2. Sleep: Neurodegenerative Disease Sleep Questionnaire (NDSQ), ESS CSF levels collection and analyses: 1. CSF was collected using a 24-gauge atraumatic needle into polypropylene tubes using a modified ADNI protocol without overnight fasting. 2. Hypocretin-1 (orexin-A) levels were measured in unextracted CSF using a highly sensitive, commercially available, 125-I radioimmunoassay kit.	1. Except for sleepiness (which is worst in DLB) and nocturia (worst in DLB and FTD), sleep symptoms did not differ by diagnosis 2. Hypocretin levels did not differ by diagnosis. 3. Lower hypocretin levels were related to frequency of nightmares (203.9 ± 29.8 pg/mL in those with frequent nightmares versus 240.4 ± 46.1 pg/mL in those without, p = 0.05) and vivid dreams (209.1 ± 28.3 in those with vivid dreams vs. 239.5 ± 47.8 pg/mL in those without; p = 0.014).	1. Reliance on patient and/or caregiver reports about sleep, rather than more objective measures. 2. Analyses did not adjust for multiple testing. 3. Dementia severity, dementia duration, and presence of REM sleep behaviour disorder might impact these findings.
Multiple System Atrophy (MSA)					
Martinez-Rodriguez et al. (2007) Barcelona, Spain Cross-sectional study	MSA	MSA: N = 15 (60.6 ± 5.4) Controls: N = 6 (54.3 ± 22.7)	Demographics and Clinical Assessments: 1. Clinical history and review 2. Disease severity: UMSARS I & II, UPDRS 3. Medication: LEDD 4. EDS: ESS MSLT PSG CSF levels collection and analyses: 1. CSF were obtained via lumbar puncture. 2. Measurements of hypocretin-1 was performed by radioimmunoassays (RIA).	1. Despite relatively low sleep efficiencies in nocturnal sleep, mean sleep latencies in MSLT were normal with no SOREM periods. 2. All patients had CSF hypocretin-1 levels in the normal range (>200 pg/mL), suggesting that the hypocretin system is not altered in MSA, at least in patients with moderately severe disease	1. Small sample size

Abbreviations: *DLB* Dementia with Lewy Bodies, *EMG* Electromyography, *EDS* Excessive Daytime Sleepiness, *ESS* Epworth Sleepiness Scale, *F* Female sample, *FTD*: Frontotemporal Dementia, *RBD* idiopathic RBD, *M* Male sample, *MMSE* Mini-Mental State Exam, *MoCA-J* Japanese version of Montreal Cognitive Assessment, *MSA* Multiple System Atrophy, *N* Total number of sample, *PD* Parkinson’s Disease, *PLM index* Periodic Limb Movement index, *PSG* Polysomnography, REM: Rapid Eye Movement, *RBD *REM Sleep Behaviour Disorder, *PSP* Progressive Supranuclear Palsy

Thirdly, it is worth highlighting that these studies have adopted different methodological approaches in terms of including the subtypes of sleep disturbances (i.e., nocturnal versus diurnal; sleep attacks, hypersomnolence, RBD), subtypes of PD (i.e., with or without dementia) and the choice of controls used or other pathological conditions [30, 62, 64, 65, 67]. On a similar note, pathological relevance of CSF orexin levels in alpha-synucleinopathies also needs to be accounted for. For instance, the benchmark for abnormal CSF orexin levels is predominantly based on diagnosis of narcolepsy (i.e., normal: >200 pg/mL, intermediate: 110 to 200 pg/mL, and abnormal: <110 pg/mL) [99]. Lastly, in most of these studies, there is also the lack of consideration of other interplaying factors that may affect the availability of orexin levels and clinical symptomology. For example, in a preclinical study, Zhan and colleagues reported that higher expression of TNF-a in neuroinflammation-induced PD mice model, is associated with the degradation of orexinergic neurons, which led to development of other non-motor symptoms and sleep disturbances [100] . Future human studies should consider utilizing a translational approach to investigate modulation of the orexinergic pathway at cellular, subcellular, and molecular level.

In summary, in keeping to those in iRBD, studies of patient with PD report diverse findings, with some stating higher, other lower than normal range, and some found normal levels of orexin in patients with PD [29, 30, 57, 61–67, 95]. Thus, to date, the reliability of orexin levels as a predictive biomarker of sleep disturbances remains unclear [29, 30, 62]. It has been similarly argued that besides potential methodological and physiological factors, these contrasting findings may reflect a complex, multifaceted pathomechanisms of the orexinergic pathway in alpha-synucleinopathies.

### Dementia with Lewy Bodies

Dementia with Lewy Bodies (DLB) is characterised by the accumulation of aggregated alpha-synuclein protein in Lewy bodies and Lewy neurites [101]). Core clinical features of DLB include neuropsychiatric symptoms, progressive cognitive decline, and parkinsonism [102]. Alpha-synuclein aggregation in orexin-containing neurons in the hypothalamus has been demonstrated [103], and various studies further reported its interference with multiple cellular functions [58, 104], potentially contributing to progressive loss of orexin-containing neurons loss in DLB. Moreover, abnormal sleep-wake rhythms, excessive daytime sleepiness, insomnia and RBD have all similarly been reported to occur in DLB [105].

Several studies have investigated orexin levels in patients with DLB [31, 54, 58, 68]. In a cross-sectional study, Trotti and colleagues examined the association of orexin, dementia biomarkers and sleep disturbances in four clinical populations: Alzheimer’s Disease (AD), frontotemporal dementia (FTD), DLB and healthy controls [54]. Here, orexin levels were predominantly associated to the type of sleep disturbances. More specifically, lower orexin-A levels were related to frequency of nightmares (patients with nightmares: 203.9 ± 29.8 pg/mL, patients without nightmares: 240.4 ± 46.1 pg/mL) and vivid dreams (209.1 ± 28.3 versus 239.5 ± 47.8 pg/mL) [54]. These findings are consistent with an earlier study by Lessig and colleagues [58] who reported reduced neocortical orexin immunoreactivity in DLB, as compared to AD patients and controls [58]. In this study, orexin levels were significantly linked with severity of hypersomnolence and alpha-synuclein levels [58], perhaps similarly suggesting that dysregulation of orexin regulating sleep-wake stability may be a more sensitive biomarker of distinct sleep phenotypes.

Conversely, other studies failed to report significant reductions in orexin levels in DLB [31, 68]. For instance, Inagawa and colleagues did not demonstrate significant differences in CSF orexin A levels between patients with DLB (271.6 ± 114.5 pg/mL) and controls (301.0 ± 64.0 pg/mL) [31]. Similarly, Baumann and colleagues showed normal levels of orexin levels in DLB (mean 521 pg/mL), as compared to controls (497 pg/mL) and AD patients (474 pg/mL) [68].

A lack of standardisation of protocols for orexin sampling, small samples sizes, different circadian rhythms, and inconsistent reporting on other potential confounding factors, such are medications and disease severity likely underlie at least some of the reported differential findings [31, 54, 58, 68].

### Multiple System Atrophy

Multiple system atrophy (MSA) is clinically characterised by varying combination of parkinsonism, pyramidal signs, and progressive autonomic failure, and/or cerebellar ataxia; it is also pathologically characterised by the abnormal accumulation of alpha-synuclein in oligodendrocytes [106, 107]. As with other alpha-synucleinopathies, sleep disturbances such as RBD, fragmented sleep, excessive daytime somnolence and nocturnal stridor are frequently reported in MSA [52, 53, 108].

Perhaps unsurprisingly given its prevalence, there are only a few studies that investigated links between orexin levels and disease severity, mostly with negative findings [52, 53]. In a small retrospective study conducted in the Netherlands, Abdo and colleagues reported that CSF orexin levels did not differ between MSA patient group (mean = 343 pg/mL) and controls (mean = 339 pg/mL) [52], although no sleep measurements were included. In keeping, Martinez-Rodriguez and colleagues reported normal CSF orexin level (>200 pg/mL) in MSA patients with significant sleep issues, suggesting that orexin levels may not be a sensitive biomarker for detecting sleep disorders in MSA [53].

The lack of clear findings is somewhat surprising given that loss of approximately 70% of orexin-producing neurons in the posterior hypothalamic regions has been reported [109]. Again, methodologic differences, small sample sizes and lack of standardisation of CSF sampling protocols (ventricular vs spinal) have been argued to underlie some of these findings, or lack of them [95].

## Discussion

We have critically reviewed the contemporary literature that investigates the orexinergic system and sleep disturbances in alpha-synucleinopathies. Our findings overwhelmingly highlight scarcity of studies that investigated the association of orexin and sleep in alpha-synucleinopathies, and further emphasise the lack of systematic and standardised methodological research protocols. Nonetheless, for iRBD and PD patients certain pattern appears to be emerging, that of an initial (compensatory) adaptive increase in orexin production, with subsequent reduction in CSF orexin levels, linked to later stages, or to more severe PD. Given that distinct alpha-synucleinopathies differ in their spatio-temporal evolution and cellular localisation, this may explain why to date, no such pattern has been reported for MSA patients [52, 53].

In striking opposition to human data, the potential role for orexin as a therapeutical intervention in alleviating sleep disturbances and other symptoms of alpha-synucleinopathies has been supported by many preclinical studies. For instance, the neuroprotective role of orexin-A on dopaminergic neurons and BDNF levels in the substantia nigra has been reported [110–115]. Nonetheless, its translational and clinical role in alpha-synucleinopathy patients is yet to be fully understood.

Also, numerous studies have reported sex-specific differences in demographics, biomarkers, and clinical features in alpha-synucleinopathies [116–118]. Coincidentally, orexin has multiple endocrine effects, including its role in the regulation of central and peripheral signals for metabolic homeostasis [32]. More specifically, orexin have been reported to inhibit the release of prolactin and growth hormone with a modulatory effect on the release of luteinizing hormone [28, 32]. Orexin also controls the hypothalamic-pituitary axis (HPA) through OX2R receptor through stimulating adrenocorticotropin (ACTH) and corticosteroid secretions via activation of cortico-trophin-releasing hormones and vasopressin [32, 119]. Notwithstanding these findings, none of the reviewed studies accounted for possible sex differences and thus, its possible theoretical interplay in alpha-synucleinopathy presentations and illness trajectories similarly remains unclear [28, 32].

Moreover, several limitations across the 17 studies that were critically assessed affected our ability to draw definitive conclusions. These limitations included small sample sizes, methodological inconsistencies, and potential biases in data collection and reporting. For instance, the variability in study designs and the lack of rigorous phenotyping in regards to a specific subtype, disease severity, possible role of medications, sleep issues/disorders and cognitive symptoms, in some cases further compromised the reliability and generalizability of the findings. Consequently, while the assessed studies provide valuable insights, their limitations necessitate cautious interpretation of the results. Hence, to obtain a more integrative and coherent framework of the role of orexinergic pathway in sleep disturbances in alpha-synucleinopathies, future studies should systematically account for (1) a specific subtype of alpha-synucleinopathies (i.e., PD with or without dementia), (2) specific sleep phenotype (i.e., sleep attacks, hypersomnolence), (3) disease progression, rate, and severity (i.e., early versus late onset), (4) the standardised collection protocol for orexin measurements, (5) circadian effect, e.g. consistency in the time of orexin measurement, (6) consideration of medications (i.e., dopaminergic treatments) and finally (7) direct quantification of sex hormones and their relation to orexin and sleep disturbances severity.

## Conclusions

Taken together, the body of work to date suggests an important, if complex and multidimensional, relationship between the dysregulation of the orexinergic pathway and the emergence of sleep disturbances in alpha-synucleinopathies. Moreover, it is possible that this interplay differentially impacts different alpha-synucleinopathies and their phenotypes, with no clear relationship so far being shown for the MSA.

Scarcity of studies and significant methodologic limitations in the current body of work disallow meaningful conclusion regarding the underlying pathomechanisms. Thus, we propose, sufficiently powered, large, longitudinal multi-centre studies of carefully phenotyped cohorts should be undertaken to obtain a more comprehensive account of the role of orexinergic pathway in sleep and other non-motor symptoms of alpha-synucleinopathies. With the new agonist and orexin receptor antagonists emerging [120], this is the perfect timing to explore therapeutic applications in treatment strategies that might be effective for treating both motor and non-motor symptoms in alpha-synucleinopathies.

## **Key References**

• P. Tall, M.A. Qamar, I. Rosenzweig, V. Raeder, A. Sauerbier, Z. Heidemarie, C. Falup- Pecurariu, and K.R. Chaudhuri, The Park Sleep subtype in Parkinson's disease: from concept to clinic. Expert Opin Pharmacother 24 (2023) 1725-1736.

      -  An updated outline of treatment strategies for improvement of disorders of sleep and wakefulness in PD patients.

• M. Alrouji, H.M. Al-Kuraishy, A.I. Al-Gareeb, D. Zaafar, and G.E.-S. Batiha, Orexin pathway in Parkinson’s disease: a review. Molecular Biology Reports (2023) 1-14.

      -  A comprehensive review of implicated orexinergic pathways in Parkinson's Disease.

• T. Ogawa, Y. Kajiyama, H. Ishido, S. Chiba, G.S. Revankar, T. Nakano, S. Taniguchi, T. Kanbayashi, K. Ikenaka, and H. Mochizuki, Decreased cerebrospinal fluid orexin levels not associated with clinical sleep disturbance in Parkinson’s disease: A retrospective study. Plos one 17 (2022) e0279747.

      -   A retrospective study that demonstrated decreased CSF orexin levels in patients with PD, but without correlation to clinical characteristics.

• H. Asai, M. Hirano, Y. Furiya, F. Udaka, M. Morikawa, T. Kanbayashi, T. Shimizu, and S. Ueno, Cerebrospinal fluid-orexin levels and sleep attacks in four patients with Parkinson's disease. Clinical Neurology and Neurosurgery 111 (2009) 341-344.

      -  A small study that was amongst first to suggest that orexin transmission is likely involved in sleep attacks in PD.

### Supplementary Information

Below is the link to the electronic supplementary material.

Supplementary file1 (DOCX 17 KB)
