# Supplementary material for: Orexin and Sleep Disturbances in Alpha-Synucleinopathies: a Systematic Review
Source: Curr Neurol Neurosci Rep. 2024 Jul 20;24(9):389–412. doi: 10.1007/s11910-024-01359-6 (PMC11349833; doi:10.1007/s11910-024-01359-6)
Supplement: Supplementary file 1 — Supplementary file1 (DOCX 17 KB) [file 11910_2024_1359_MOESM1_ESM.docx]

**SUPPLEMENT**

**SS Table 1:** Methodological evaluation of studies using the EPHPP Quality Assessment Tool for Quantitative Studies

| **Author and Year** | **Disease** | **Selection Bias** | **Study Design** | **Confounders** | **Blinding** | **Data Collection** | **Withdrawals and Drop-outs** | **Global rating** |
| --- | --- | --- | --- | --- | --- | --- | --- | --- |
| **Anderson et al., (2010)** | RBD | Moderate | Moderate | Strong | Moderate | Strong | N/A | Strong |
| **Yuan et al., (2022)** | RBD | Moderate | Moderate | Strong | Moderate | Strong | N/A | Strong |
| **Wienecke et al., (2012)** | PD | Moderate | Moderate | Moderate | Moderate | Strong | N/A | Moderate |
| **Ogawa et al., (2022)** | PD | Moderate | Moderate | Moderate | Moderate | Strong | N/A | Moderate |
| **Drout et al., (2003)** | PD | Weak | Moderate | Weak | Moderate | Strong | N/A | Weak |
| **Afdal et al., (2021)** | PD | Strong | Moderate | Weak | Moderate | Strong | N/A | Moderate |
| **Huang et al., (2021)** | PD | Moderate | Moderate | Strong | Moderate | Strong | N/A | Strong |
| **Takahashi et al., (2015)** | PD | Weak | Moderate | Weak | Moderate | Strong | N/A | Weak |
| **Bridoux et al., (2013)** | PD | Weak | Moderate | Weak | Moderate | Strong | N/A | Weak |
| **Asai et al., (2009)** | PD | Moderate | Moderate | Weak | Moderate | Strong | N/A | Moderate |
| **Compta et al., (2009)** | PD | Strong | Moderate | Strong | Moderate | Strong | N/A | Strong |
| **Yasui et al., (2006)** | PD | Moderate | Moderate | Moderate | Moderate | Strong | N/A | Strong |
| **Inagawa et al., (2021)** | DLB | Moderate | Moderate | Weak | Moderate | Strong | N/A | Moderate |
| **Lessig et al., (2010)** | DLB | Weak | Moderate | Weak | Moderate | Strong | N/A | Weak |
| **Baumann et al., (2004)** | DLB | Moderate | Moderate | Moderate | Moderate | Strong | N/A | Strong |
| **Trotti et al., (2021)** | DLB | Moderate | Moderate | Weak | Moderate | Strong | N/A | Moderate |
| **Martinez-Rodriguez et al., (2007)** | MSA | Moderate | Moderate | Weak | Moderate | Strong | N/A | Moderate |

**Abbreviations**: **DLB**: Dementia with Lewy Bodies; **MSA**: Multiple System Atrophy; **PD**: Parkinson’s Disease; **RBD**: REM Sleep Behaviour Disor
